# Supplementary material for: A database study of clinical and economic burden of invasive meningococcal disease in France
Source: PLoS One. 2022 Apr 29;17(4):e0267786. doi: 10.1371/journal.pone.0267786 (PMC9053794; doi:10.1371/journal.pone.0267786)
Supplement: S1 Table — (DOCX) [file pone.0267786.s001.docx]

**S1 Table. ICD-10 codes used to identify the study population**

| **ICD-10 codes** | **Meningococcemia only** |
| --- | --- |
| **IMD** |  |
| A39 | Meningococcal infection |
| A39.0 | Meningococcal meningitis |
| A39.1 | Waterhouse-Friderichsen syndrome |
| A39.2 | Acute meningococcaemia |
| A39.4 | Meningococcaemia, unspecified |
| A39.5 | Meningococcal heart disease |
| A39.8 | Other meningococcal infections |
| A39.9 | Meningococcal infections, unspecified |
| **IMD’s manifestations** |  |
| E351 | Disorders of adrenal glands in diseases classified elsewhere |
| G01 | Meningitis in bacterial diseases classified elsewhere |
| G050 | Encephalitis, myelitis and encephalomyelitis in bacterial diseases classified elsewhere |
| H131 | Conjunctivitis in infectious and parasitic diseases classified elsewhere |
| H481 | Retrobulbar neuritis in diseases classified elsewhere |
| I320 | Pericarditis in bacterial diseases classified elsewhere |
| I398 | Endocarditis, valve unspecified, in diseases classified elsewhere |
| I410 | Myocarditis in bacterial diseases classified elsewhere |
| I520 | Other heart disorders in bacterial diseases classified elsewhere |
| M010 | Meningococcal arthritis |

International Statistical Classification of Diseases and Related Health Problem 10^th^ Revision
