# Supplementary material for: A database study of clinical and economic burden of invasive meningococcal disease in France
Source: PLoS One. 2022 Apr 29;17(4):e0267786. doi: 10.1371/journal.pone.0267786 (PMC9053794; doi:10.1371/journal.pone.0267786)
Supplement: S2 Table — (DOCX) [file pone.0267786.s002.docx]

**S2 Table. ICD-10 codes used to identify IMD-related sequelae**

| **IMD-related sequelae** | **Algorithm (combination of ICD-10 and procedures codes)** |
| --- | --- |
| **Neurological sequelae** | At least one of the following CCAM or ICD-10 codes (as main, related or associated diagnosis within MCO or SSR records) during the follow-up (including the index stay), excluding those with at least one of these codes in the 12 months before index date: |
| Motor deficit | **ICD-10 codes for isolated hypotonia, motor delay, ataxia, gait or coordination difficulties:**   - F82 Specific developmental disorder of motor function - R26 Abnormalities of gait and mobility   - R26.0 Ataxic gait   - R26.1 Paralytic gait   - R26.2 Difficulty in walking, not elsewhere classified   - R26.3 Immobility   - R26.8 Other and unspecified abnormalities of gait and mobility - R27 Other lack of coordination   - R27.0 Ataxia, unspecified   - R27.8 Other and unspecified lack of coordination - G24 Dystonia   - G24.9 Dystonia, unspecified - G25 Other extrapyramidal and movement disorders   - G25.3 Myoclonus   - G25.5 Other chorea   - G25.9 Extrapyramidal and movement disorder, unspecified   **ICD-10 codes for impairment, spasticity or paresis of one or more limbs:**   - G80 Cerebral palsy   - G80.0 Spastic quadriplegic cerebral palsy   - G80.1 Spastic diplegic cerebral palsy   - G80.2 Spastic hemiplegic cerebral palsy   - G80.3 Dyskinetic cerebral palsy   - G80.4 Ataxic cerebral palsy   - G80.8 Other cerebral palsy   - G80.9 Cerebral palsy, unspecified - G81 Hemiplegia   - G81.0 Flaccid hemiplegia   - G81.1 Spastic hemiplegia   - G81.9 Hemiplegia, unspecified - G82 Paraplegia and tetraplegia   - G82.0 Flaccid paraplegia   - G82.1 Spastic paraplegia   - G82.2 Paraplegia, unspecified   - G82.3 Flaccid tetraplegia   - G82.4 Spastic tetraplegia   - G82.5 Tetraplegia, unspecified - G83 Other paralytic syndromes   - G83.0 Diplegia of upper limbs   - G83.1 Monoplegia of lower limb   - G83.2 Monoplegia of upper limb   - G83.3 Monoplegia, unspecified |
| Seizure | **ICD-10 codes**:   - G40 Epilepsy - G41 Status epilepticus |
| Visual disturbance | **ICD-10 codes:**   - H47 Other disorders of optic [2nd] nerve and visual pathways - H49 Paralytic strabismus - H50 Other strabismus - H51 Other disorders of binocular movement - H53 Visual disturbances   - H53.0 Amblyopia ex anopsia   - H53.1 Subjective visual disturbances   - H53.2 Diplopia   - H53.3 Other disorders of binocular vision   - H53.4 Visual field defects   - H53.5 Colour vision deficiencies   - H53.6 Night blindness   - H53.8 Other visual disturbances   - H53.9 Visual disturbance, unspecified - H54 Visual impairment including blindness (binocular or monocular)   - H54.0 Blindness, binocular   - H54.1 Severe visual impairment, binocular   - H54.2 Moderate visual impairment, binocular   - H54.3 Mild or no visual impairment, binocular   - H54.4 Blindness, monocular   - H54.5 Severe visual impairment, monocular   - H54.6 Moderate visual impairment, monocular   - H54.9 Unspecified visual impairment (binocular) |
| Hydrocephalus | **ICD-10 codes**:   - G91 Hydrocephalus   - G91.0 Communicating hydrocephalus   - G91.1 Obstructive hydrocephalus   - G91.2 Normal-pressure hydrocephalus   - G91.8 Other hydrocephalus   - G91.9 Hydrocephalus, unspecified   **ICD-10 codes for cerebrospinal fluid drainage devices:**   - Z98.2 Presence of cerebrospinal fluid drainage device - T85.0 Mechanical complication of ventricular intracranial (communicating) shunt   **CCAM^^[[1]](#footnote-1)^^ codes for cerebrospinal fluid drainage:**   - *ABCA001 Ventriculoventriculostomie, ventriculocisternostomie, kystocisternostomie ou kystoventriculostomie, par craniotomie* - *ABCA002 Dérivation péritonéale ou atriale du liquide cérébrospinal ventriculaire, par abord direct* - *ABCA003 Dérivation péritonéale d'une collection subdurale du liquide cérébrospinal crânien, par abord direct* - *ABCB001 Dérivation externe du liquide cérébrospinal ventriculaire ou subdural, par voie transcrânienne* - *ABCC001 Ventriculoventriculostomie, ventriculocisternostomie,kystocisternostomie ou kystoventriculostomie, par vidéochirurgie intracrânienne* - *ABMA003 Révision ou changement d'élément d'une dérivation interne du liquide cérébrospinal, par craniotomie* - *AFJB002 Évacuation de liquide cérébrospinal, par voie transcutanée lombale* |
| **Auditive Impairement** | At least one of the following CCAM or ICD-10 codes (as main, related or associated diagnosis within MCO or SSR records) during the follow-up (including the index stay), excluding those with at least one of these codes in the 12 months before index date: |
|  | **ICD-10 codes**:   - H90.3 Sensorineural hearing loss, bilateral - H90.4 Sensorineural hearing loss, unilateral with unrestricted hearing on the contralateral side - H90.5 Sensorineural hearing loss, unspecified - H90.6 Mixed conductive and sensorineural hearing loss, bilateral - H90.7 Mixed conductive and sensorineural hearing loss, unilateral with unrestricted hearing on the contralateral side - H90.8 Mixed conductive and sensorineural hearing loss, unspecified - H91.8 Other specified hearing loss - H91.9 Hearing loss, unspecified   **ICD-10 codes** **for implanted neuroprosthetic devices:**   - Z45.3 Adjustment and management of implanted hearing device - Z96.2 Presence of otological and audiological implants   **CCAM procedure codes for implantation of neuroprosthetic devices:**   - *CDLA002 Pose d'un implant auditif à électrodes du tronc cérébral* - *CDLA003 Pose d'un implant auditif à électrodes intracochléaires* - *CDLA004 Pose d'un implant auditif à électrodes extracochléaires* |
| **Cognitive impairement** | At least one of the following ICD-10 codes (as main, related or associated diagnosis within MCO or SSR records) during the follow-up (including the index stay), excluding those with at least one of these codes in the 12 months before index date: |
| Cognitive impairement | **ICD-10 codes**:   - F06.7 Mild cognitive disorder - F80.0 Specific speech articulation disorder   - F80.1 Expressive language disorder   - F80.2 Receptive language disorder   - F80.3 Acquired aphasia with epilepsy [Landau-Kleffner]   - F80.8 Other developmental disorders of speech and language   - F80.9 Developmental disorder of speech and language, unspecified - F81 Specific developmental disorders of scholastic skills   - F81.0 Specific reading disorder   - F81.1 Specific spelling disorder   - F81.2 Specific disorder of arithmetical skills   - F81.3 Mixed disorder of scholastic skills   - F81.8 Other developmental disorders of scholastic skills   - F81.9 Developmental disorder of scholastic skills, unspecified |
| Mental retardation | **ICD-10 codes**:   - F06.7 Mild cognitive disorder - F70-F79 Mental retardation |
| Behavioural disorder | **ICD-10 codes**:   - F90-98 Behavioral disorder |
| **Chronic renal failure** | At least one of the following CCAM or ICD-10 codes (as main, related or associated diagnosis within MCO or SSR records) during the follow-up (including the index stay), excluding those with at least one of these codes in the 12 months before index date: |
|  | **ICD-10 codes:**   - N18 Chronic kidney disease   **CCAM codes for dialysis for chronic renal failure:**   - *JVJF003 Séance d'épuration extrarénale par hémoperfusion* - *JVJF004 Séance d'épuration extrarénale par hémodialyse pour insuffisance rénale chronique* - *JVJF008 Séance d'épuration extrarénale par hémodiafiltration, hémofiltration ou biofiltration sans acétate pour insuffisance rénale chronique* - *JVJB001 Séance d'épuration extrarénale par dialyse péritonéale pour insuffisance rénale chronique* - *JVRP004 Séance d'entraînement à l'hémodialyse* - *JVRP007 Séance d'entraînement à la dialyse péritonéale automatisée* - *JVRP008 Séance d'entraînement à la dialyse péritonéale continue ambulatoire* |
| **Amputation/ Skin necrosis** | At least one of the following CCAM or ICD-10 codes (as main, related or associated diagnosis within MCO or SSR records) during the follow-up (including the index stay), excluding those with at least one of these codes in the 12 months before index date: |
| Amputation/ limb loss | **ICD-10 codes**:   - S48 Traumatic amputation of shoulder and upper arm - S58 Traumatic amputation of forearm - S68 Traumatic amputation of wrist and hand - S78 Traumatic amputation of hip and thigh - S88 Traumatic amputation of lower leg - S98 Traumatic amputation of ankle and foot - T05 Traumatic amputations involving multiple body regions - T13.6 Traumatic amputation of lower limb, level unspecified - Z89 Acquired absence of limb   **CCAM procedure codes for amputation of upper limb:**   - *MZFA001 Amputation complète d'un rayon de la main* - *MZFA002 Amputation transhumérale* - *MZFA003 Amputation complète de plusieurs rayons de la main* - *MZFA005 Amputation transradio-ulnaire* - *MZFA007 Amputation et/ou désarticulation de plusieurs doigts, sans résection des métacarpiens* - *MZFA013 Amputation ou désarticulation d'un doigt, sans résection du métacarpien*   **CCAM procedure codes for amputation of lower limb:**   - *NZFA002 Amputation transtibiale* - *NZFA004 Amputation ou désarticulation de plusieurs orteils* - *NZFA005 Amputation ou désarticulation au médiopied ou à l'avant-pied, sans stabilisation de l'arrière-pied* - *NZFA007 Amputation transfémorale* - *NZFA009 Amputation ou désarticulation à la cheville ou à l'arrière-pied* - *NZFA010 Amputation ou désarticulation d'un orteil* - *NZFA013 Amputation ou désarticulation du médiopied ou de l'avant-pied, avec stabilisation de l'arrière-pied* |
| Skin necrosis/skin grafting | **ICD-10 codes**:   - R02 Gangrene, not elsewhere classified - M72.6 Necrotizing fasciitis   **ICD-10 codes for skin grafting**:   - Z94.5 Skin transplant status   **CCAM procedure codes for skin grafting:**   - *QZEA001 Greffe cutanée en sandwich, sur plus de 20% de la surface corporelle* - *QZEA002 Greffe cutanée pour brûlure en dehors de l'extrémité céphalique et des mains, sur 15% à 17,5% de la surface corporelle* - *QZEA003 Greffe de culture de kératinocytes autologues pour brûlure, sur 30% à 40% de la surface corporelle* - *QZEA004 Greffe cutanée en sandwich, sur 5% à 7,5% de la surface corporelle* - *QZEA005 Autogreffe de peau mince ou demiépaisse, pleine ou en filet, sur une surface de 500 cm² à 1000 cm²* - *QZEA006 Autogreffe de peau totale sur plusieurs localisations* - *QZEA008 Autogreffe du lit de l'ongle avec reposition de la tablette unguéale ou pose de prothèse* - *QZEA009 Relèvement de 6 cicatrices ou plus, par abord direct* - *QZEA010 Greffe cutanée pour brûlure en dehors de l'extrémité céphalique et des mains, sur plus de 20% de la surface corporelle* - *QZEA011 Greffe cutanée pour brûlure en dehors de l'extrémité céphalique et des mains, sur 10% à 12,5% de la surface corporelle* - *QZEA012 Greffe cutanée pour brûlure en dehors de l'extrémité céphalique et des mains, sur 12,5% à 15% de la surface corporelle* - *QZEA013 Greffe cutanée en sandwich, sur 7,5% à 10% de la surface corporelle* - *QZEA014 Greffe cutanée en sandwich, sur 12,5% à 15% de la surface corporelle* - *QZEA016 Greffe de culture de kératinocytes autologues pour brûlure, sur 40% à 50% de la surface corporelle* - *QZEA017 Greffe cutanée pour brûlure en dehors de l'extrémité céphalique et des mains, sur 7,5% à 10% de la surface corporelle* - *QZEA019 Autogreffe de peau en pastilles sur une surface de 10 cm² à 50 cm²* - *QZEA020 Autogreffe de peau mince ou demiépaisse, pleine ou en filet, sur une surface inférieure à 50 cm²* - *QZEA021 Greffe cutanée pour brûlure en dehors de l'extrémité céphalique et des mains, sur moins de 2,5% de la surface corporelle* - *QZEA022 Greffe de culture de kératinocytes autologues pour brûlure, sur 20% à 30% de la surface corporelle* - *QZEA023 Greffe de culture de kératinocytes autologues pour brûlure, sur moins de 10% de la surface corporelle* - *QZEA024 Autogreffe de peau totale sur une localisation de surface égale ou supérieure à 10 cm²* - *QZEA025 Greffe cutanée pour brûlure en dehors de l'extrémité céphalique et des mains, sur 17,5% à 20% de la surface corporelle* - *QZEA026 Autogreffe de peau en pastilles sur une surface supérieure à 50 cm²* - *QZEA027 Autogreffe de peau mince ou demiépaisse, pleine ou en filet, sur une surface de 200 cm² à 500 cm²* - *QZEA028 Transfert libre de complexe unguéomatriciel avec anastomoses vasculaires* - *QZEA029 Greffe cutanée en sandwich, sur 2,5% à 5% de la surface corporelle* - *QZEA030 Greffe cutanée en sandwich, sur 17,5% à 20% de la surface corporelle* - *QZEA031 Autogreffe de peau totale sur une localisation de surface inférieure à 10 cm²* - *QZEA032 Autogreffe de peau en pastilles sur une surface inférieure à 10 cm²* - *QZEA033 Greffe de culture de kératinocytes autologues pour brûlure, sur plus de 50% de la surface corporelle* - *QZEA034 Relèvement de 1 à 5 cicatrices, par abord direct* - *QZEA036 Autogreffe de peau mince ou demiépaisse, pleine ou en filet, sur une surface supérieure à 1000 cm²* - *QZEA037 Greffe cutanée pour brûlure en dehors de l'extrémité céphalique et des mains, sur 2,5% à 5% de la surface corporelle* - *QZEA038 Greffe cutanée en sandwich, sur moins de 2,5% de la surface corporelle* - *QZEA039 Autogreffe de peau mince ou demiépaisse, pleine ou en filet, sur une surface de 50 cm² à 200 cm²* - *QZEA040 Greffe cutanée en sandwich, sur 15% à 17,5% de la surface corporelle* - *QZEA041 Greffe cutanée pour brûlure en dehors de l'extrémité céphalique et des mains, sur 5% à 7,5% de la surface corporelle* - *QZEA042 Greffe de culture de kératinocytes autologues pour brûlure, sur 10% à 20% de la surface corporelle* - *QZEA043 Greffe cutanée en sandwich, sur 10% à 12,5% de la surface corporelle* - *QZEA044 Autogreffe souscutanée susfasciale de peau désépidermisée pour comblement de dépression cutanée, par abord direct* - *QZEA045 Autogreffe souscutanée susfasciale de tissu celluloadipeux pour comblement de dépression cutanée, par abord direct* - *QZEA900 Autogreffe de mélanocytes de culture* - *QZEA901 Autogreffe de mélanocytes de transfert* - *QZLB001 Injection souscutanée susfasciale de tissu adipeux* |
| **Arthritis** | At least one of the following ICD-10 codes (as main, related or associated diagnosis within MCO or SSR records) during the follow-up (including the index stay), excluding those with at least one of these codes in the 12 months before index date: |
|  | **ICD-10 codes**:   - M01.0 Meningococcal arthritis (A39.8) *(code also used for ascertaining clinical presentation of IMD, to be identified during the follow-up,* ***excluding index stay****)* - M03.0 Postmeningococcal arthritis |

1. [↑](#footnote-ref-1)
