# Supplementary material for: A database study of clinical and economic burden of invasive meningococcal disease in France
Source: PLoS One. 2022 Apr 29;17(4):e0267786. doi: 10.1371/journal.pone.0267786 (PMC9053794; doi:10.1371/journal.pone.0267786)
Supplement: Attachment — Submitted filename: Plos One Rebuttal 24 Sept 2021 CLEAN.docx [file pone.0267786.s003.docx]

Rebuttal PLOS One

| Journal Requirements:  When submitting your revision, we need you to address these additional requirements.  1. Please ensure that your manuscript meets PLOS ONE's style requirements, including those for file naming. The PLOS ONE style templates can be found at <https://journals.plos.org/plosone/s/file?id=wjVg/PLOSOne_formatting_sample_main_body.pdf> and <https://journals.plos.org/plosone/s/file?id=ba62/PLOSOne_formatting_sample_title_authors_affiliations.pdf> |  |
| --- | --- |
| 2. We note that the grant information you provided in the ‘Funding Information’ and ‘Financial Disclosure’ sections do not match.  When you resubmit, please ensure that you provide the correct grant numbers for the awards you received for your study in the ‘Funding Information’ section. | For clarity, we had deleted the financial information from acknowledgement.  We had amended the financial disclosure in the system too.  The “Acknowledgement” now reads as below  *We would like to thank to CASD – Centre d’accès sécurisé aux données (Ref. 10.34724/CASD) for making accessible to some confidential data within a secured environment.* |
| 3. Thank you for providing the following Funding Statement:  “This project was funded by Pfizer France. LH, SF, AE and MTTH are employees of Pfizer. They had a role in study design, decision to publish and preparation of the manuscript.  SB, LM and MG were paid by Pfizer France as a consultancy company for study design, data collection in analysis, and development of the manuscript.”  We note that one or more of the authors is affiliated with the funding organization, indicating the funder may have had some role in the design, data collection, analysis or preparation of your manuscript for publication; in other words, the funder played an indirect role through the participation of the co-authors.  If the funding organization did not play a role in the study design, data collection and analysis, decision to publish, or preparation of the manuscript and only provided financial support in the form of authors' salaries and/or research materials, please review your statements relating to the author contributions, and ensure you have specifically and accurately indicated the role(s) that these authors had in your study in the Author Contributions section of the online submission form.  Please make any necessary amendments directly within this section of the online submission form.  Please also update your Funding Statement to include the following statement: “The funder provided support in the form of salaries for authors [insert relevant initials], but did not have any additional role in the study design, data collection and analysis, decision to publish, or preparation of the manuscript. The specific roles of these authors are articulated in the ‘author contributions’ section.”  If the funding organization did have an additional role, please state and explain that role within your Funding Statement.  Please also provide an updated Competing Interests Statement declaring this commercial affiliation along with any other relevant declarations relating to employment, consultancy, patents, products in development, or marketed products, etc. | Competing interests and financial disclosures are amended as below.  Competing interests: we need a statement form Pfizer publication team for Pfizer authors. L Huang, A Elkaim, S Fievez, M Tin Tin Htar are employees of Pfizer Inc. and hold company shares. L Marie, M Goguillot, and S Bénard are employees of Stève consultants, Oullins, France and this work was funded by Pfizer.  Financial disclosure: The analysis was fully funded by Pfizer Inc. LH, AE, SF and MTTH are Pfizer employees and they took part in the design of the study, analysis, and interpretation of data and in writing the manuscript. |
| Within your Competing Interests Statement, please confirm that this commercial affiliation does not alter your adherence to all PLOS ONE policies on sharing data and materials by including the following statement: "This does not alter our adherence to PLOS ONE policies on sharing data and materials.” (as detailed online in our guide for authors http://journals.plos.org/plosone/s/competing-interests). If this adherence statement is not accurate and there are restrictions on sharing of data and/or materials, please state these. Please note that we cannot proceed with consideration of your article until this information has been declared. | We, Pfizer authors, confirm that our affiliation to the Pfizer commercial industry do not alter the adherence to PLOS ONE policies on sharing data and materials. |
| 4. In your Data Availability statement, you have not specified where the minimal data set underlying the results described in your manuscript can be found. PLOS defines a study's minimal data set as the underlying data used to reach the conclusions drawn in the manuscript and any additional data required to replicate the reported study findings in their entirety. All PLOS journals require that the minimal data set be made fully available. For more information about our data policy, please see <http://journals.plos.org/plosone/s/data-availability>.  "Upon re-submitting your revised manuscript, please upload your study’s minimal underlying data set as either Supporting Information files or to a stable, public repository and include the relevant URLs, DOIs, or accession numbers within your revised cover letter. For a list of acceptable repositories, please see http://journals.plos.org/plosone/s/dataavailability#loc-recommended-repositories. Any potentially identifying patient information must be fully anonymized.  Important: If there are ethical or legal restrictions to sharing your data publicly, please explain these restrictions in detail. Please see our guidelines for more information on what we consider unacceptable restrictions to publicly sharing data: http://journals.plos.org/plosone/s/data-availability#loc-unacceptable-data-access-restrictions. Note that it is not acceptable for the authors to be the sole named individuals responsible for ensuring data access.  We will update your Data Availability statement to reflect the information you provide in your cover letter. | The underlying dataset could not be made publicly available. Aggregated data are set in appropriate tables in the results session. |
| 5. Please review your reference list to ensure that it is complete and correct. If you have cited papers that have been retracted, please include the rationale for doing so in the manuscript text, or remove these references and replace them with relevant current references. Any changes to the reference list should be mentioned in the rebuttal letter that accompanies your revised manuscript. If you need to cite a retracted article, indicate the article’s retracted status in the References list and also include a citation and full reference for the retraction notice. | The list is correct. |
| Reviewers' comments:  Reviewer's Responses to Questions  **Comments to the Author**  1. Is the manuscript technically sound, and do the data support the conclusions? The manuscript must describe a technically sound piece of scientific research with data that supports the conclusions. Experiments must have been conducted rigorously, with appropriate controls, replication, and sample sizes. The conclusions must be drawn appropriately based on the data presented.  Reviewer #1: Yes | Thank you |
| 2. Has the statistical analysis been performed appropriately and rigorously?  Reviewer #1: Yes | Thank you |
| 3. Have the authors made all data underlying the findings in their manuscript fully available? The PLOS Data policy requires authors to make all data underlying the findings described in their manuscript fully available without restriction, with rare exception (please refer to the Data Availability Statement in the manuscript PDF file). The data should be provided as part of the manuscript or its supporting information, or deposited to a public repository. For example, in addition to summary statistics, the data points behind means, medians and variance measures should be available. If there are restrictions on publicly sharing data—e.g. participant privacy or use of data from a third party—those must be specified. References list and also include a citation and full reference for the retraction notice.  [NOTE: If reviewer comments were submitted as an attachment file, they will be attached to this email and accessible via the submission site. Please log into your account, locate the manuscript record, and check for the action link "View Attachments". If this link does not appear, there are no attachment files.] While revising your submission, please upload your figure files to the Preflight Analysis and Conversion Engine (PACE) digital diagnostic tool, https://pacev2.apexcovantage.com/. PACE helps ensure that figures meet PLOS requirements. To use PACE, you must first register as a user. Registration is free. Then, login and navigate to the UPLOAD tab, where you will find detailed instructions on how to use the tool. If you encounter any issues or have any questions when using PACE, please email PLOS at figures@plos.org. Please note that Supporting Information files do not need this step.  Reviewer #1: Yes | Thank you |
| 4. Is the manuscript presented in an intelligible fashion and written in standard English? PLOS ONE does not copyedit accepted manuscripts, so the language in submitted articles must be clear, correct, and unambiguous. Any typographical or grammatical errors should be corrected at revision, so please note any specific errors here.  Reviewer #1: Yes | Thank you |
| 5. Review Comments to the Author Please use the space provided to explain your answers to the questions above. You may also include additional comments for the author, including concerns about dual publication, research ethics, or publication ethics. (Please upload your review as an attachment if it exceeds 20,000 characters) |  |
| Reviewer #1: Thanks so much for inviting me to review this manuscript. It is well written and very interesting paper. Vaccination has greatly reduced the burden of infectious diseases. It is a double edged sword. Vaccine-preventable diseases have gone down, followed by a rise in vaccine hesitancy. It is great to read a paper investigating the burden of IMD, a life threatening disease. I only have a few minor comments.  Financial disclosure (page 1): In the Acknowledgement section, the authors stated the work was supported by a public grant. This grant was not mentioned here and it was only said that the project was funded by Pfizer. | The research was not a public grant but funded by Pfizer. We had amended accordingly.  The section has been updated as below.  Financial disclosure: The analysis was fully funded by Pfizer Inc. LH, AE, SF and MTTH are Pfizer employees and they took part in the design of the study, analysis, and interpretation of data and in writing the manuscript. |
| Introduction  Line 31: The authors stated that case fatality rate (CRF) ranged from 10% to 40%. Only in elderly people, the CFR could reach 40%. On average, the CFR ranged from 4-20%. | Thank you. We have added “being the highest rate observed in elderly” to the text. |
| Statistical Analysis  Line 102: can the author please add more information and explain why GLM with gamma distribution and a log link function was used? Did the authors conduct any model fitting tests? | This specific model was used due to the distribution of costs (non negative and tend to be skewed to the right). No model fitting tests was performed due to the objective of this analyses (descriptive and non-predictive)  This now reads in the statistical analysis session as bellow.  Multivariate analysis was conducted in patients with at least one year of follow-up to examine factors associated with costs during the first year of IMD. Due to the statistical distribution of costs (costs are non-negative and tend to be skewed to the right), this analysis was derived from a generalized linear model with gamma distribution and a log link function. No model fitting tests was performed. |
| Results  Lines 117/120/126: Error messages. Can the authors remove or revise the references? | Updated in the manuscript but without tracked changes |
| Table 2: Amputation/skin necrosis. I would expect amputation and skin necrosis could all be diagnosed during the index hospitalisation. But, 16 patients with amputation and skin necrosis were identified after the index hospitalisation. I suspect a few skin grafting procedures might be performed after the index hospitalisation. If that was true, can the authors please add skin grafting? | Yes. 16 additional patients underwent amputation/skin necrosis after discharge from index hospitalization.  As specified in the S2 Table, hospitalizations associated with skin necrosis and amputation have been identified via ICD-10 codes and CCAM (French classification of medical procedures). A code of skin grafting was included in this list.  We have added as requested the “skin grafting” in the table 2 and in the text whenever necessary. |
| Line 155: Would it better to say numbers (16 of 23) rather than percentages (1.2% of 1.7%)? | Thank you. We have amended to (16 of 23) |
| Table 4: The annualised costs were reported. Can the authors please explain in the statistical analysis section how the annualised costs were calculated? | The had added the explanation in the statistical analysis as below.    “These annualized costs have been calculated as average costs adjusted to the follow-up period and meant to compare costs estimated on different follow-up periods, by adjusting them by the follow-up period of patients.” |
| Table 5: The total number of patients with at least one-year follow up is 792. However, Table 4 says 760 had at least one year follow up time. Can the authors explain why 32 patients were not included in Table 4? | Thank you.  Thank you. There are 792 patients with ≥12 months of follow-up. However, in the table 3, “13- ≤24 months” does not include those with follow-up period of 12 months.  Actually, 32 patients were not included in the table 4 in the 13-24 months category since they have exactly 12 months.  Then We had amended the text in results session to be aligned with the table 3 as below.  “Of them, 1,019 (80.6%) had at least more than 7 months of follow-up time, 760 (60.1%) had at least 13 months, and 340 (26.9%) had at least 25 months of follow-up time.” |
| Discussion  Line 270: survivors? | Thank you. We have edited accordingly. |
| Limitations: Serogroups could not be included in the analysis. Were outpatient visits included? For IMD cases with limb disabilities, speech problems and chronic kidney impairments, physiotherapy, speech therapy and dialysis costs could be high as well. | No. Outpatients visits are not included. Only all-cause hospitalization and rehabilitation costs are included. Database that has been used only reports hospitalisations data, including day-hospitalisation (dialysis). Unfortunately, outpatient visits were not available in the database. |
| 6. PLOS authors have the option to publish the peer review history of their article (what does this mean?). If published, this will include your full peer review and any attached files. | We decline this option. |
